# Correntropy Based Divided Difference Filtering for the Positioning of Ships

**DOI:** 10.3390/s18114080

**Published:** 2018-11-21

**Authors:** Xi Liu, Badong Chen, Shiyuan Wang, Shaoyi Du

**Affiliations:** 1 School of Electronic and Information Engineering, Xi’an Jiaotong University, Xi’an 710049, China; lx1102@stu.xjtu.edu.cn (X.L.); dushaoyi@mail.xjtu.edu.cn (S.D.); 2 College of Electronic and Information Engineering, Southwest University, Chongqing 400715, China; wsy@swu.edu.cn

**Keywords:** divided difference filter (DDF), correntropy, Dead Reckoning (DR), Global Positioning System (GPS)

## Abstract

In this paper, robust first and second-order divided difference filtering algorithms based on correntropy are proposed, which not only retain the advantages of divided difference filters, but also exhibit robustness in the presence of non-Gaussian noises, especially when the measurements are contaminated by heavy-tailed noises. The proposed filters are then applied to the problem of ship positioning. In order to improve the accuracy and reliability of ship positioning, the positioning method combines the Dead Reckoning (DR) algorithm and the Global Positioning System (GPS). Experimental results of an illustrative example show the superior performance of the new algorithms when applied to ship positioning.

## 1. Introduction

With the development of microcomputers and electronic integration technology, the requirements for accuracy and reliability of ship positioning are becoming more and more complex. As a key node in the air-space-ground of integrated information networks, ships can obtain the positioning information from many systems. The Dead Reckoning (DR) is a common technology used in ship positioning, which calculates the position of ship in real time based on the speed of ship and direction information, it also considers the influence of wind and flow. However, errors in the speed and direction information, and incomplete compensations of wind and flow cause positioning errors of DR which accumulate with time [1,2]. Global Positioning System (GPS) is a satellite based system, which provides the accurate velocity and position of a ship by making use of the GPS receiver. But GPS has limitations such as a low sampling rate as well as being susceptible to interference [3,4,5]. The integration of DR and GPS takes advantage of two techniques, this integration system has a better performance than the single techniques in terms of accuracy and reliability [6,7,8].

Filtering is a key problem in the integrated positioning system. The Kalman filter (KF) [9,10] is a well known method to estimate the state of linear systems. However, the model of DR/GPS integrated positioning system is nonlinear. To solve the nonlinear filtering problem, extensions of the KF using some approximations have been proposed. The extended Kalman filter (EKF) [11,12,13] approximates the nonlinear equation by its first-order linearization. The unscented Kalman filter (UKF) [13,14,15,16] approximates the probability distribution of the state by a set of deterministically chosen sigma points and propagates the distribution through the nonlinear equation, which provides higher-order approximation than the EKF. Nevertheless, the parameters used in the UKF have a great effect on performance of the algorithm. If they are not tuned finely, it is easy to face the problem of numerical instability in practical applications due to the propagation of the non-positive definite covariance matrix. Another effective, alternative way is the divided difference filter (DDF). The DDF [17,18] is derived from polynomial approximations of the nonlinear transformations using multidimensional Stirling’s interpolation formula and can be classified into the first-order divided difference (DD1) filter and the second-order divided difference (DD2) filter. The DDF can guarantee a positive semi-definiteness of the covariance matrix. The DD2 filter not only has fewer parameters, but also has nearly the same performance as the UKF. Therefore, we use the DDF type filters for ship positioning. The traditional DDF is suitable for the Gaussian noise. However, in this real application, the measuring instruments can be affected by extreme sea environments. They sometimes break down, or suffer from operator error, which cause the measurement noise to be of the heavy-tailed non-Gaussian form. In these cases, the traditional DDF, which is based on the minimum mean square error (MMSE) criterion, cannot play well because the MMSE criterion is very sensitive to the heavy-tailed non-Gaussian noise [19].

To solve the non-Gaussian filtering problems, some robust algorithms exist. The Huber methodology is a method that combines minimum $\ell_{1}$ and $\ell_{2}$-norm [20,21,22]. The Student *t* technique assumes that the process and measurement noises obey Student *t* distribution [23,24]. Another effective approach is the information theoretic learning (ITL). In particular, the correntropy, which is one of the ITL measures, can capture high-order statistics of the data rather than the common second-order statistics and has been widely used in many fields [25,26,27,28,29,30,31,32]. In recent years, some correntropy-based Kalman filterings have been proposed [33,34,35], which are mainly applied to linear models. The correntropy-based unscented Kalman filters can solve some nonlinear problems [36,37], but it is easy to have problems with numerical instability for integrated positioning.

In this paper, two novel nonlinear filters, the correntropy-based first-order divided difference (CDD1) filter and the correntropy-based second-order divided difference (CDD2) filter, are proposed to solve the problem of ship positioning. The proposed algorithms not only retain the advantages of DDF algorithms, but also exhibit the robustness in the presence of heavy-tailed non-Gaussian noise. Different from the works [38] which adopts the linear regression model, the proposed algorithms adopt the nonlinear regression model.

The rest of the paper is organized as follows. Section 2 provides a short review of the correntropy, the DD1, and DD2 filters. In Section 3, the the CDD1 and CDD2 filters are derived. In Section 4, the example of ship positioning shows the performance of the proposed algorithms. The conclusion is given in Section 5.

## 2. Preliminaries

### 2.1. Correntropy

The correntropy is an important measure in ITL, which measures the similarity between two random variables X∈R and Y∈R. Given the joint distribution function of *X* and *Y*, $F_{XY}\left(x,y\right)$, the correntropy is defined by

(1) $$V\left(X,Y\right)=E\left[\kappa (X,Y)\right]=\int \kappa \left(x,y\right)dF_{XY}\left(x,y\right)$$

where E[·] represents the expectation operator, and κ(·,·) is a shift-invariant Mercer kernel. In this paper, the Gaussian kernel is chosen as the kernel function:

(2) $$\kappa \left(x,y\right)=G_{\sigma }\left(e\right)=\exp\left(-\frac{e^{2}}{2\sigma^{2}}\right)$$

where e=x−y, and σ>0 denotes the kernel bandwidth.

In most practical applications, only a limited number of data samples are available and the joint distribution $F_{XY}$ is usually unknown. In this case, we often use the sample mean estimator to estimate the correntropy:

(3) $$\hat{V}\left(X,Y\right)=\frac{1}{N}\sum_{i=1}^{N}G_{\sigma }\left(e(i)\right)$$

where e(i)=x(i)−y(i), with ${\left\{x(i),y(i)\right\}}_{i=1}^{N}$ being *N* samples drawn from $F_{XY}$.

Taking the Taylor series expansion for the Gaussian kernel yields

(4) $$V\left(X,Y\right)=\sum_{n=0}^{\infty }\frac{{\left(-1\right)}^{n}}{2^{n}\sigma^{2n}n!}E\left[{\left(X-Y\right)}^{2n}\right]$$

Note that the correntropy is the weighted sum of all even order moments of the error variable X−Y and the kernel bandwidth appears as a parameter to weight the second-order and higher-order moments. In particular, with a very large kernel bandwidth, the second-order moment will play a major role.

### 2.2. DD1 Filter

In this paper, the nonlinear state and measurement equations are described by

(5) $$x_{k}=f\left(x_{k-1},q_{k-1}\right)$$

(6) $$y_{k}=h\left(x_{k},r_{k}\right)$$

where $x\left(k\right)\in R{}^{n}$ and $y\left(k\right)\in R{}^{m}$ are the *n*-dimensional state vector and *m*-dimensional measurement vector at time step *k*. $f\left(\cdot \right)$ and $h\left(\cdot \right)$ are the continuous and differentiable state functions and the measurement function. $q_{k-1}$ and $r_{k}$ are the process and measurement noises, which are assumed i.i.d and independent of states, and with means denoted by ${\bar{q}}_{k-1}$ and ${\bar{r}}_{k}$ and covariance matrices denoted by $Q_{k-1}$ and $R_{k}$.

The square-root decompositions of the predicted state error covariance ${\bar{P}}_{k}$, update state error covariance ${\hat{P}}_{k}$, process noise covariance $Q_{k}$, and measurement noise covariance $R_{k}$ are introduced as

(7) $${\bar{P}}_{k}={\bar{S}}_{x_{k}}{\bar{S}}_{x_{k}}^{T}$$

(8) $${\hat{P}}_{k}={\hat{S}}_{x_{k}}{\hat{S}}_{x_{k}}^{T}$$

(9) $$Q_{k}=S_{q_{k}}S_{q_{k}}^{T}$$

(10) $$R_{k}=S_{r_{k}}S_{r_{k}}^{T}$$

The DD1 filter uses the first-order divided differences to approximate. Let the element of *i*-th row, *j*-th column of ${S^{\prime }}_{x{\hat{x}}_{k}}$ be denoted as ${S^{\prime }}_{x{\hat{x}}_{k}i,j}$, i.e., ${S^{\prime }}_{x{\hat{x}}_{k}}=\left\{{S^{\prime }}_{x{\hat{x}}_{k}i,j}\right\}$, and similarly for other matrices. The four matrices containing first-order divided differences are defined as

(11) S′xx^k−1=fix^k−1+cS^xk−1,j,q¯k−1−fix^k−1−cS^xk−1,j,q¯k−1fix^k−1+cS^xk−1,j,q¯k−1−fix^k−1−cS^xk−1,j,q¯k−12c2c

(12) S′xqk−1=fix^k−1,q¯k−1+cSqk−1,j−fix^k−1,q¯k−1−cSqk−1,jfix^k−1,q¯k−1+cSqk−1,j−fix^k−1,q¯k−1−cSqk−1,j2c2c

(13) S′yx¯k=hix¯k+cS¯xk,j,r¯k−hix¯k−cS¯xk,j,r¯khix¯k+cS¯xk,j,r¯k−hix¯k−cS¯xk,j,r¯k2c2c

(14) S′yrk=hix¯k,r¯k+cSrk,j−hix¯k,r¯k−cSrk,jhix¯k,r¯k+cSrk,j−hix¯k,r¯k−cSrk,j2c2c

where ${\hat{S}}_{x_{k-1},j}$ is the *j*-th column of ${\hat{S}}_{x_{k-1}}$, *c* is the interval length, and is generally set as $c^{2}=3$.

The predicted state and the Cholesky factor of corresponding covariance are given by

(15) $${\bar{x}}_{k}=f\left({\hat{x}}_{k-1},{\bar{q}}_{k-1}\right)$$

(16) $${\bar{S}}_{x_{k}}=H\left(\left[\begin{array}{cc} {S^{\prime }}_{x{\hat{x}}_{k-1}} & {S^{\prime }}_{xq_{k-1}} \end{array}\right]\right)$$

where $H\left(\cdot \right)$ denotes a Householder transformation of the augment matrix [17].

The predicted measurement and the Cholesky factor of corresponding covariance are given by

(17) $${\bar{y}}_{k}=h\left({\bar{x}}_{k},{\bar{r}}_{k}\right)$$

(18) $$S_{y_{k}}=H\left(\left[\begin{array}{cc} {S^{\prime }}_{y{\bar{x}}_{k}} & {S^{\prime }}_{yr_{k}} \end{array}\right]\right)$$

The Kalman gain is computed as

(19) $$K_{k}={\bar{S}}_{x_{k}}{S^{\prime }}_{y{\bar{x}}_{k}}^{T}{\left(S_{y_{k}}S_{y_{k}}^{T}\right)}^{-1}$$

The updated state and the Cholesky factor of corresponding covariance are given by

(20) $${\hat{x}}_{k}={\bar{x}}_{k}+K_{k}\left(y_{k}-{\bar{y}}_{k}\right)$$

(21) $${\hat{S}}_{x_{k}}=H\left(\left[\begin{array}{cc} {\bar{S}}_{x_{k}}-K_{k}{S^{\prime }}_{y{\bar{x}}_{k}} & K_{k}{S^{\prime }}_{yr_{k}} \end{array}\right]\right)$$

### 2.3. DD2 Filter

The DD2 filter uses the second-order divided differences to approximate. Four matrices containing second-order divided differences are defined as

(22) $$\begin{array}{cc} {S^{\prime \prime }}_{x{\hat{x}}_{k-1}}= & \left\{\frac{\sqrt{c^{2}-1}}{2c^{2}}\left[f_{i}\left({\hat{x}}_{k-1}+c{\hat{S}}_{x_{k-1},j},{\bar{q}}_{k-1}\right)+f_{i}\left({\hat{x}}_{k-1}-c{\hat{S}}_{x_{k-1},j},{\bar{q}}_{k-1}\right)\right.\right.\;\\ & \left.\left.-2f_{i}\left({\hat{x}}_{k-1},{\bar{q}}_{k-1}\right)\right]\right\} \end{array}$$

(23) $$\begin{array}{cc} {S^{\prime \prime }}_{xq_{k-1}}= & \left\{\frac{\sqrt{c^{2}-1}}{2c^{2}}\right.\left[f_{i}\left({\hat{x}}_{k-1},{\bar{q}}_{k-1}+cS_{q_{k-1},j}\right)\right.+f_{i}\left({\hat{x}}_{k-1},{\bar{q}}_{k-1}-cS_{q_{k-1},j}\right)\;\\ & \left.\left.-2f_{i}\left({\hat{x}}_{k-1},{\bar{q}}_{k-1}\right)\right]\right\} \end{array}$$

(24) $$\begin{array}{cc} {S^{\prime \prime }}_{y{\bar{x}}_{k}}= & \left\{\frac{\sqrt{c^{2}-1}}{2c^{2}}\left[h_{i}\left({\bar{x}}_{k}+c{\bar{S}}_{x_{k},j},{\bar{r}}_{k}\right)\right.\right.+h_{i}\left({\bar{x}}_{k}-c{\bar{S}}_{x_{k},j},{\bar{r}}_{k}\right)\;\\ & \left.\left.-2h_{i}\left({\bar{x}}_{k},{\bar{r}}_{k}\right)\right]\right\} \end{array}$$

(25) $$\begin{array}{cc} {S^{\prime \prime }}_{yr_{k}}= & \left\{\frac{\sqrt{c^{2}-1}}{2c^{2}}\left[h_{i}\left({\bar{x}}_{k},{\bar{r}}_{k}+cS_{r_{k},j}\right)\right.\right.+h_{i}\left({\bar{x}}_{k},{\bar{r}}_{k}-cS_{r_{k},j}\right)\\ & \left.\left.-2h_{i}\left({\bar{x}}_{k},{\bar{r}}_{k}\right)\right]\right\} \end{array}$$

The predicted state and the Cholesky factor of corresponding covariance are given by

(26) $$\begin{array}{cc} {\bar{x}}_{k}= & \left(\frac{c^{2}-n_{x}-n_{q}}{c^{2}}\right)f\left({\hat{x}}_{k-1},{\bar{q}}_{k-1}\right)\\ & +\frac{1}{2c^{2}}\sum_{j=1}^{n_{x}}\left[f\left({\hat{x}}_{k-1}+c{\hat{S}}_{x_{k-1},j},{\bar{q}}_{k-1}\right)+f\left({\hat{x}}_{k-1}-c{\hat{S}}_{x_{k-1},j},{\bar{q}}_{k-1}\right)\right]\\ & +\frac{1}{2c^{2}}\sum_{j=1}^{n_{q}}\left[f\left({\hat{x}}_{k-1},{\bar{q}}_{k-1}+cS_{q_{k-1},j}\right)+f\left({\hat{x}}_{k-1},{\bar{q}}_{k-1}-cS_{q_{k-1},j}\right)\right] \end{array}$$

(27) $${\bar{S}}_{x_{k}}=H\left(\left[\begin{array}{cccc} {S^{\prime }}_{x{\hat{x}}_{k-1}} & {S^{\prime }}_{xq_{k-1}} & {S^{\prime \prime }}_{x{\hat{x}}_{k-1}} & {S^{\prime \prime }}_{xq_{k-1}} \end{array}\right]\right)$$

The predicted measurement and the Cholesky factor of corresponding covariance are given by

(28) $$\begin{array}{cc} {\bar{y}}_{k}= & \left(\frac{c^{2}-n_{x}-n_{r}}{c^{2}}\right)h\left({\bar{x}}_{k},{\bar{r}}_{k}\right)\\ & +\frac{1}{2c^{2}}\sum_{j=1}^{n_{x}}\left[h\left({\bar{x}}_{k}+c{\bar{S}}_{x_{k},j},{\bar{r}}_{k}\right)+h\left({\bar{x}}_{k}-c{\bar{S}}_{x_{k},j},{\bar{r}}_{k}\right)\right]\\ & +\frac{1}{2c^{2}}\sum_{j=1}^{n_{r}}\left[h\left({\bar{x}}_{k},{\bar{r}}_{k}+cS_{r_{k},j}\right)+h\left({\bar{x}}_{k},{\bar{r}}_{k}-cS_{r_{k},j}\right)\right] \end{array}$$

(29) $$S_{y_{k}}=H\left(\left[\begin{array}{cccc} {S^{\prime }}_{y{\bar{x}}_{k}} & {S^{\prime }}_{yr_{k}} & {S^{\prime \prime }}_{y{\bar{x}}_{k}} & {S^{\prime \prime }}_{yr_{k}} \end{array}\right]\right)$$

The Kalman gain is computed as

(30) $$K_{k}={\bar{S}}_{x_{k}}{S^{\prime }}_{y{\bar{x}}_{k}}^{T}{\left(S_{y_{k}}S_{y_{k}}^{T}\right)}^{-1}$$

The updated state and the Cholesky factor of corresponding covariance are given by

(31) $${\hat{x}}_{k}={\bar{x}}_{k}+K_{k}\left(y_{k}-{\bar{y}}_{k}\right)$$

(32) $${\hat{S}}_{x_{k}}=H\left(\left[\begin{array}{cccc} {\bar{S}}_{x_{k}}-K_{k}{S^{\prime }}_{y{\bar{x}}_{k}} & K_{k}{S^{\prime }}_{yr_{k}} & K_{k}{S^{\prime \prime }}_{y{\bar{x}}_{k}} & K_{k}{S^{\prime \prime }}_{yr_{k}} \end{array}\right]\right)$$

## 3. Correntropy-Based DDF

This section derives the DD1 and DD2 filters under maximum correntropy criterion (MCC). First, the measurement Equation (6), whose noise is additive, would be written as $y_{k}=h\left(x_{k}\right)+r_{k}$, and the predicted state and the Choleskey factor of corresponding covariance are obtained by Equations (15) and (16). Assuming the true state is denoted as $x_{k}$, the predicted state error is written as $\xi_{k}=x_{k}-{\bar{x}}_{k}$. Then, a nonlinear regression model is reconstructed as follows:

(33) $$\left[\begin{array}{c} {\bar{x}}_{k}\\ y_{k} \end{array}\right]=\left[\begin{array}{c} x_{k}\\ h(x_{k}) \end{array}\right]+\delta_{k}$$

where $\delta_{k}=\left[\begin{array}{c} -\xi_{k}\\ r_{k} \end{array}\right]$, with the Cholesky factor of covariance of $\delta_{k}$ is given by

(34) $$D_{k}=\left[\begin{array}{cc} {\bar{S}}_{x_{k}} & 0\\ 0 & S_{r_{k}} \end{array}\right]$$

Left multiplying both sides of Equation (33) by $D_{k}^{-1}$, we have the following regression model:

(35) $$z_{k}=m\left(x_{k}\right)+e_{k}$$

where $z_{k}=D_{k}^{-1}\left[\begin{array}{c} {\bar{x}}_{k}\\ y_{k} \end{array}\right]$, $m\left(x_{k}\right)=D_{k}^{-1}\left[\begin{array}{c} x_{k}\\ h\left(x_{k}\right) \end{array}\right]$ and $e_{k}=D_{k}^{-1}\delta_{k}$.

The problem associated with Equation (35) can be solved by making use of the MCC, and the corresponding cost function is given by

(36) $$J\left(x_{k}\right)=\sum_{i=1}^{L}G_{\sigma }\left(e_{k,i}\right)$$

where $e_{k,i}$ is the *i*-th component of the vector $e_{k}=z_{k}-m\left(x_{k}\right)$, and *L* is the dimension of $e_{k}$.

Then, the solution of the problem mentioned previously can be found by setting the first derivation of Equation (36) to be zero:

(37) $$\sum_{i=1}^{L}\phi \left(e_{k,i}\right)\frac{\partial e_{k,i}}{\partial x_{k}}=0$$

where $\phi \left(e_{k,i}\right)=G_{\sigma }\left(e_{k,i}\right)\cdot e_{k,i}$. By defining Cek,i=ϕek,iϕek,iek,i=Gσek,i=Gσek,i and $C=diag\left[C\left(e_{k,i}\right)\right]=\left[\begin{array}{cc} C_{x} & 0\\ 0 & C_{y} \end{array}\right]$, Equation (37) can be written in matrix form as

(38) $${\left(\frac{\partial m\left(x_{k}\right)}{\partial x_{k}}\right)}^{T}C\left(m\left(x_{k}\right)-z_{k}\right)=0$$

In fact, the MCC uses C to re-weight the covariance matrix of the residual error $e_{k}$ and reconstruct the measurement information. Thus, the updated covariance can be written as

(39) $$\tilde{\Psi }=D_{k}C^{-1}D_{k}^{T}$$

and decomposed into two portion so that

(40) $$\tilde{\Psi }=\left[\begin{array}{cc} {\tilde{\Psi }}_{x} & 0\\ 0 & {\tilde{\Psi }}_{y} \end{array}\right]$$

The initial value can be set as ${\hat{x}}_{k}^{\left(0\right)}={\bar{x}}_{k}$ or be equal to the updated state computed from the corresponding standard DD1 or DD2. Then, we can obtain the following equations

(41) $${\tilde{\Psi }}_{x}={\bar{S}}_{x_{k}}\cdot C_{x}^{-1}\cdot {\bar{S}}_{x_{k}}^{T}$$

(42) $${\tilde{\Psi }}_{y}=S_{r_{k}}\cdot C_{y}^{-1}\cdot S_{r_{k}}^{T}$$

It is noted that the aforementioned derivation is the extension of Equation (25) in Reference [33]. To reduce the computation time, only one iteration would work to obtain the solution.

Then, a one-step correntropy update for the DD1 filter can be written as

(43) $$S_{y_{k}}^{\left(1\right)}=H\left(\left[\begin{array}{cc} {S^{\prime }}_{y{\bar{x}}_{k}}C_{x}^{-1/2} & {S^{\prime }}_{yr_{k}}C_{y}^{-1/2} \end{array}\right]\right)$$

(44) $$K_{k}^{\left(1\right)}={\bar{S}}_{x_{k}}C_{x}^{-1}{S^{\prime }}_{y{\bar{x}}_{k}}^{T}{\left(S_{y_{k}}^{\left(1\right)}S_{y_{k}}^{(1)T}\right)}^{-1}$$

(45) $${\hat{x}}_{k}={\bar{x}}_{k}+K_{k}^{\left(1\right)}\left(y_{k}-{\bar{y}}_{k}\right)$$

(46) $${\hat{S}}_{x_{k}}=H\left(\left[\begin{array}{cc} {\bar{S}}_{x_{k}}C_{x}^{-1/2}-K_{k}^{\left(1\right)}{S^{\prime }}_{y{\bar{x}}_{k}}C_{x}^{-1/2} & K_{k}^{\left(1\right)}{S^{\prime }}_{yr_{k}}C_{y}^{-1/2} \end{array}\right]\right)$$

Similarly, a one-step correntropy update for the DD2 filter can be written as

(47) $$S_{y_{k}}^{\left(1\right)}=H\left(\left[\begin{array}{cccc} {S^{\prime }}_{y{\bar{x}}_{k}}C_{x}^{-1/2} & {S^{\prime }}_{yr_{k}}C_{y}^{-1/2} & {S^{\prime \prime }}_{y{\bar{x}}_{k}}C_{x}^{-1/2} & {S^{\prime \prime }}_{yr_{k}}C_{y}^{-1/2} \end{array}\right]\right)$$

(48) $$K_{k}^{\left(1\right)}={\bar{S}}_{x_{k}}C_{x}^{-1}{S^{\prime }}_{y{\bar{x}}_{k}}^{T}{\left(S_{y_{k}}^{\left(1\right)}S_{y_{k}}^{(1)T}\right)}^{-1}$$

(49) $${\hat{x}}_{k}={\bar{x}}_{k}+K_{k}^{\left(1\right)}\left(y_{k}-{\bar{y}}_{k}\right)$$

(50) $$\begin{array}{cc} {\hat{S}}_{x_{k}}= & H\left(\left[\begin{array}{cc} {\bar{S}}_{x_{k}}C_{x}^{-1/2}-K_{k}^{\left(1\right)}{S^{\prime }}_{y{\bar{x}}_{k}}C_{x}^{-1/2} & K_{k}^{\left(1\right)}{S^{\prime }}_{yr_{k}}C_{y}^{-1/2} \end{array}\right.\right.\\ & \left.\left.\begin{array}{cc} K_{k}^{\left(1\right)}{S^{\prime \prime }}_{y{\bar{x}}_{k}}C_{x}^{-1/2} & K_{k}^{\left(1\right)}{S^{\prime \prime }}_{yr_{k}}C_{y}^{-1/2} \end{array}\right]\right) \end{array}$$

**Remark** **1.** *The proposed correntropy-based divided difference filters utilize the correntropy theory to improve the performance in the presence of heavy-tailed non-Gaussian noises, in which a nonlinear regression problem is introduced to update the measurement information. It is noted that the kernel bandwidth plays a key role in the proposed algorithm. In general, a smaller kernel bandwidth exhibits more robust properties of the correntropy. However, when the kernel bandwidth is too small, it will lead to an accretion of estimation error or even filtering divergence. A sufficient condition for guaranteeing the convergence of filter was introduced in Reference [39]. Moreover, when kernel bandwidth σ→∞, the matrix C→I, and the CDD1 and CDD2 filters would reduce to the original DD1 and DD2 filters. The choice of the kernel bandwidth in practical applications is discussed in next section.*

In practical applications, the measurement system may sometimes obtain extremely large measurements. In this case, the CDD1 and CDD2 filters may face numerical problems since $C_{y}$ will be nearly singular. In view of this problem, a method is introduced as

(51) $$a_{k}={\left(y_{k}-{\bar{y}}_{k}\right)}^{T}{\left(S_{y_{k}}S_{y_{k}}^{T}\right)}^{-1}\left(y_{k}-{\bar{y}}_{k}\right)$$

If $\left|a_{k}\right|>\theta$, with θ being a positive threshold, only the predicted step is worked, that is ${\hat{x}}_{k}={\bar{x}}_{k}$, ${\hat{S}}_{x_{k}}={\bar{S}}_{x_{k}}$. If $\left|a_{k}\right|⩽\theta$, the entire steps are worked.

**Figure 1:**
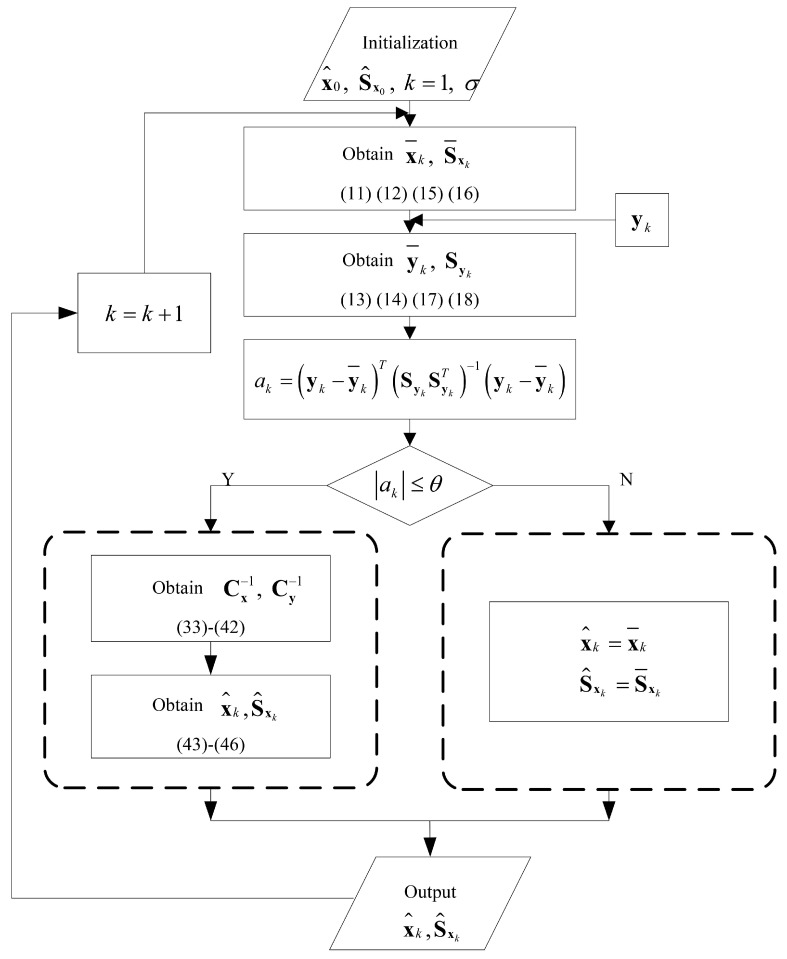
The flow of the CDD1 filter algorithm.

The flow of the CDD1 filter algorithm is shown in Figure 1. Since the flow of the CDD2 filter is similar, we omit it.

## 4. Positioning of Ships

In this section, to demonstrate the performance of the proposed algorithms, we apply them to solve the problem of ship positioning. The model combines DR and GPS technology to improve the accuracy of positioning.

### 4.1. The State and Measurement Models

The motion of a ship can be denoted as a nonlinear function in terms of many factors, such as speed, course, shape of earth, sea current, wind, and so on. There are two kinds of maneuvering motions of a ship, which are speed maneuver in a straight line and direction maneuver. Since the acceleration of the ship is generally small and the sampling period of the integrated positioning system is relatively short, the speed maneuver can be ignored or regarded as the speed noise. The direction maneuver can be approximately described by uniform circular motion with a constant change rate of the ship’s course. Therefore, there are two kinds of states related to the motion of a ship, which are uniform linear motion and uniform circular motion. In view of the above, the state vector is chosen as

$$x={\left[\begin{array}{ccccccc} \varphi & \lambda & v_{N} & v_{E} & s & K & \Omega \end{array}\right]}^{T}$$

where φ and λ denote the arc lengths of latitude and longitude, $v_{N}$ and $v_{E}$ are the northward velocity component and the eastward velocity component of the ocean current, *s* denotes the velocity of that ship relative to the water, *K* is the ship’s course, and Ω represents the change rate of the ship’s course. Correspondingly, the state equations of the ship are written as [40]

(52) $$\dot{\varphi }=v_{N}+s\cos K+q_{1}$$

(53) $$\dot{\lambda }=v_{E}+s\sin K+q_{2}$$

(54) $${\dot{v}}_{N}=-\beta v_{N}+q_{3}$$

(55) $${\dot{v}}_{E}=-\beta v_{E}+q_{4}$$

(56) $$\dot{s}=q_{5}$$

(57) $$\dot{K}=\Omega +q_{6}$$

(58) $$\dot{\Omega }=q_{7}$$

where β denotes the inverse correlation time of ocean current, $q_{i}$ are independent Gaussian white noises. By discretizing these equations, we obtain the following equations

(59) $$\begin{array}{cc} \varphi_{k}=\; & \varphi_{k-1}+\beta^{-1}\left(1-\exp(-\beta T)\right)v_{N,k-1}+\\ & s_{k-1}\cos\left(K_{k-1}+0.5T\Omega_{k-1}\right)T+q_{1,k-1} \end{array}$$

(60) $$\begin{array}{cc} \lambda_{k}=\; & \lambda_{k-1}+\beta^{-1}\left(1-\exp(-\beta T)\right)v_{E,k-1}+\\ & s_{k-1}\sin\left(K_{k-1}+0.5T\Omega_{k-1}\right)T+q_{2,k-1} \end{array}$$

(61) $$v_{N,k}=\exp\left(-\beta T\right)v_{N,k-1}+q_{3,k-1}$$

(62) $$v_{E,k}=\exp\left(-\beta T\right)v_{E,k-1}+q_{4,k-1}$$

(63) $$s_{k}=s_{k-1}+q_{5}$$

(64) $$K_{k}=K_{k-1}+\Omega_{k-1}T+q_{6,k-1}$$

(65) $$\Omega_{k}=\Omega_{k-1}+q_{7,k-1}$$

where *T* is sampling period.

Then, the measurement vector is chosen as

$$y_{k}={\left[\begin{array}{cccc} \varphi_{G,k} & \lambda_{G,k} & s_{L,k} & K_{G,k} \end{array}\right]}^{T}$$

where $\varphi_{G}$ and $\lambda_{G}$ denote the arc lengths of latitude and longitude provided by GPS, $s_{L}$ is the velocity of that ship relative to the water provided by electromagnetic log, $K_{G}$ represents the ship’s course provided by electric gyrocompass. Accordingly, the measurement equations are given as

(66) $$\varphi_{G,k}=\varphi_{k}+\eta_{1,k}$$

(67) $$\lambda_{G,k}=\lambda_{k}+\eta_{2,k}$$

(68) $$s_{L,k}=s_{k}+\eta_{3,k}$$

(69) $$K_{G,k}=K_{k}+\eta_{4,k}$$

where $\eta_{i,k}$ are independent white noises.

Some filters are used for comparison, including the EKF, DD1, UKF, DD2, the Huber-based first-order divided difference (HDD1) filter and the Huber-based second-order divided difference (HDD2) filter, and the HDD1 and HDD2 adopt the Huber methodology. The following benchmarks are used to show the estimation performance:

(70) $${\mathrm{MSE}}_{k}=\frac{1}{M}\sum_{k=1}^{M}{\left(x_{k}-{\hat{x}}_{k}\right)}^{2}$$

(71) $$\mathrm{TMSE}=\frac{1}{k_{2}-k_{1}+1}\sum_{k=k_{1}}^{k_{2}}{\mathrm{MSE}}_{k}$$

In this simulation, 100 independent Monte Carlo experiments were conducted, and the lasting time of each experiment was 1200 s. The parameters of this example and initial conditions are summarized in Table 1 and Table 2, and the process noises satisfy the Gaussian distributions:

$$\begin{array}{c} q_{1}∼N\left(0,\;0.684{\;m}^{2}\right),\;q_{2}∼N\left(0,\;0.684{\;m}^{2}\right)\;\\ q_{3}∼N\left(0,\;0.000158\;{\left(m/s\right)}^{2}\right),\;q_{4}∼N\left(0,\;0.000158\;{\left(m/s\right)}^{2}\right)\;\\ q_{5}∼N\left(0,\;0.00158\;{\left(m/s\right)}^{2}\right),\;q_{6}∼N\left(0,\;0.0026\;\mathrm{ra}d^{2}\right)\;\\ q_{7}∼0 \end{array}$$

### 4.2. Simulation Results

First, we assume the measurement noises satisfy the Gaussian distributions:

$$\begin{array}{c} r_{1}∼N\left(0,\;10000{\;m}^{2}\right),\;r_{2}∼N\left(0,\;10000\;m^{2}\right)\;\\ r_{3}∼N\left(0,\;0.0423\;{\left(m/s\right)}^{2}\right),\;r_{4}∼N\left(0,\;0.0000395\;\mathrm{ra}d^{2}\right) \end{array}$$

TMSEs of position in Gaussian noises are revealed in Table 3. It can be seen that the DD2 filter and UKF have a similar performance, likewise for the DD1 filter and EKF. The DD2 filter and UKF are superior to the DD1 filter and EKF, exhibiting smaller errors. Meanwhile, the robust filters do not perform as well as their non-robust counterparts under Gaussian noise conditions. Moreover, the CDD1 and CDD2 filters achieve almost the same performance as the DD1 and DD2 filters when the kernel bandwidth is large enough. It is noted that the UKF sometimes stops executing because the parameters of the UKF may not be finely tuned enough to bring about the problem of propagation of the non-positive definite covariance matrix.

Second, we assume the measurement noises are heavy-tailed non-Gaussian, satisfying the following distributions:

$$\begin{array}{c} r_{1}∼N\left(0,\;10000\;m^{2}\right)+N\left(0,\;1000000\;m^{2}\right)\;\\ r_{2}∼N\left(0,\;10000\;m^{2}\right)+N\left(0,\;1000000\;m^{2}\right)\;\\ r_{3}∼N\left(0,\;0.0423\;{\left(m/s\right)}^{2}\right)+N\left(0,4.23\;{\left(m/s\right)}^{2}\right)\;\\ r_{4}∼N\left(0,\;0.0000395\;\mathrm{ra}d^{2}\right)+N\left(0,\;0.00395\;\mathrm{ra}d^{2}\right) \end{array}$$

**Figure 2:**
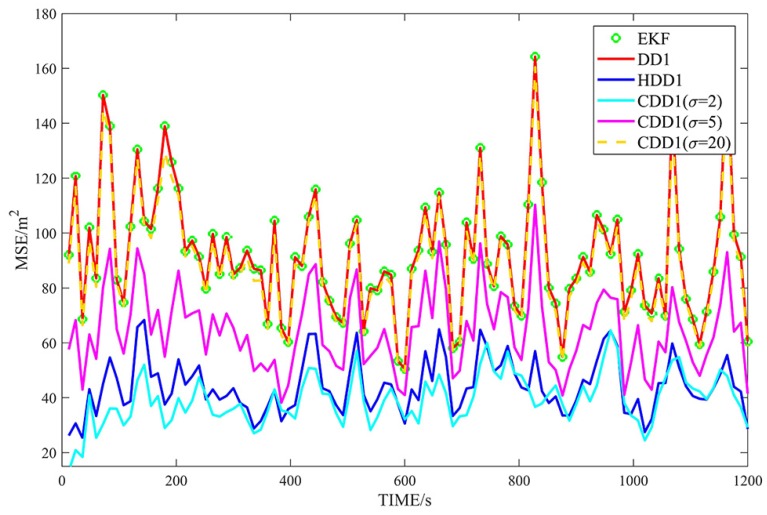
MSEs of φ with first-order approximate filters in non-Gaussian noises.

**Figure 3:**
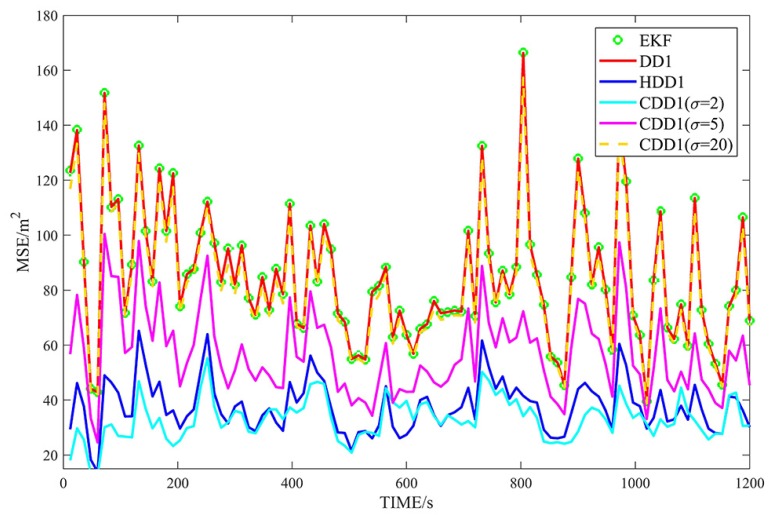
MSEs of λ with first-order approximate filters in non-Gaussian noises.

**Figure 4:**
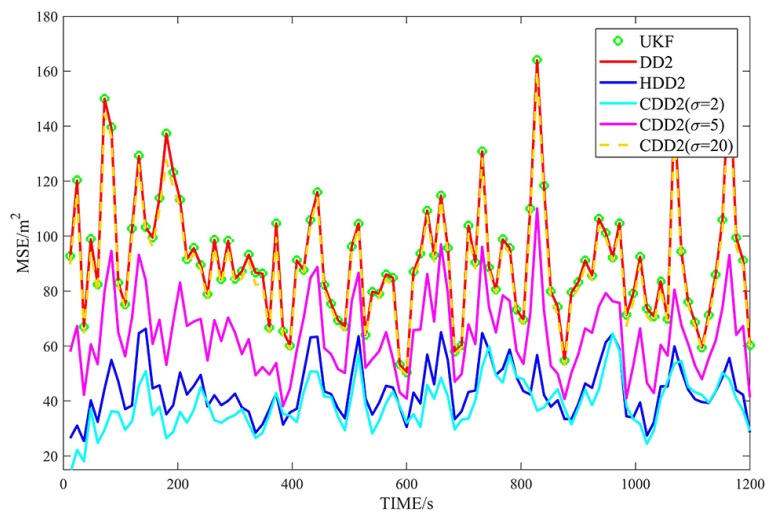
MSEs of φ with second-order approximate filters in non-Gaussian noises.

**Figure 5:**
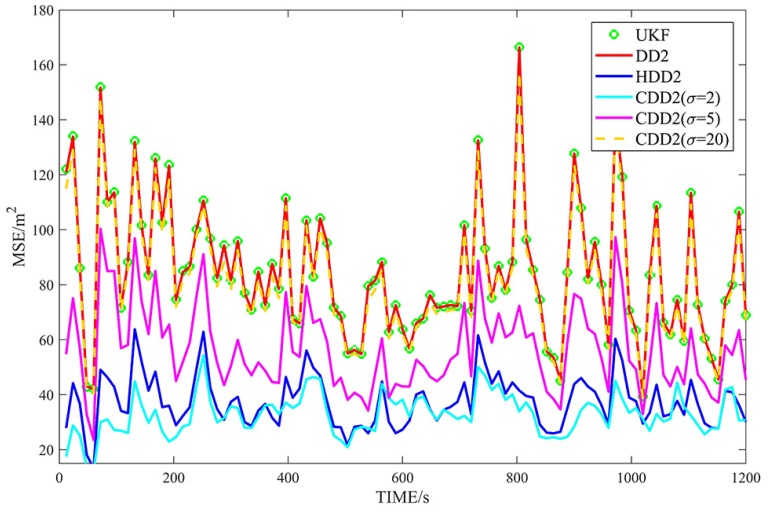
MSEs of λ with second-order approximate filters in non-Gaussian noises.

Figure 2, Figure 3, Figure 4 and Figure 5 show the MSEs of different filters for the non-Gaussian case, and Table 4 summarizes the corresponding TMSEs. The performances of the DD2, UKF, DD1, and EKF follow the behavior from the Gaussian case. In the non-Gaussian case, the robust filters outperform the corresponding non-robust filters. With a very large kernel bandwidth, the CDD1 and CDD2 filters achieve a similar estimation to the DD1 and DD2 filters; with a proper kernel bandwidth, the CDD1 and CDD2 give smaller errors than the non-robust filters; in particular, when the kernel bandwidth is set to 2, the CDD2 exhibits the smallest errors.

## 5. Conclusions

This paper proposes two correntropy-based divided difference filtering methods, namely CDD1 and CDD2, which show strong robustness against heavy-tailed non-Gaussian noises. The proposed algorithms recast the nonlinear regression models and use the maximum correntropy criterion to obtain the solution. The two robust filters are then applied to the DR/GPS integrated positioning system of ships. The filters used for comparison include the EKF, DD1, HDD1, UKF, DD2, and HDD2. The results show that with a very large kernel bandwidth, the performances of the CDD1 and CDD2 filters are similar to those of standard DD1 and DD2 filters; with a proper kernel bandwidth, the CDD2 filter exhibits the best performance for the non-Gaussian noise case. Moreover, extensions of this research might include combining it with adaptive filtering methods, considering the problem of continuous systems, and applying it to other practical examples.

## Figures and Tables

**Table 1:** Parameters of the example.

Parameter	Value
$\beta^{-1},s$	27,780/*s*
T,s	12

**Table 2:** Initial conditions.

Parameter	Value
$\varphi_{0},m$	$2.2239\times 10^{6}$
$\lambda_{0},m$	$1.2565\times 10^{7}$
$v_{N,0},m/s$	1
$v_{E,0},m/s$	1
$s_{0},m/s$	10.289
$K_{0},\mathrm{rad}$	π/4
$\Omega_{0},\mathrm{rad}/s$	0

**Table 3:** TMSEs of position in Gaussian noises.

Filter	TMSE **s** **of** φ	TMSE **s** **of** λ
EKF	10.3197	11.1708
DD1	10.3196	11.1707
HDD1	12.1968	13.1074
CDD1 $\left(\sigma =2\right)$	23.8353	22.6151
CDD1 $\left(\sigma =5\right)$	10.4517	11.2693
CDD1 $\left(\sigma =20\right)$	10.3215	11.1690
UKF	10.3093	11.1557
DD2	10.3086	11.1549
HDD2	12.1613	13.0604
CDD2 $\left(\sigma =2\right)$	23.8503	22.3792
CDD2 $\left(\sigma =5\right)$	10.4366	11.2484
CDD2 $\left(\sigma =20\right)$	10.3102	11.1529

**Table 4:** TMSEs of position in non-Gaussian noises.

Filter	TMSE **s** **of** φ	TMSE **s** **of** λ
EKF	91.7839	84.7678
DD1	91.7776	84.7557
HDD1	44.2747	37.6608
CDD1 $\left(\sigma =2\right)$	39.4284	33.0989
CDD1 $\left(\sigma =5\right)$	64.5026	56.9180
CDD1 $\left(\sigma =20\right)$	89.3531	82.3220
UKF	91.4480	84.5617
DD2	91.4269	84.5294
HDD2	43.8700	37.4722
CDD2 $\left(\sigma =2\right)$	39.0126	32.8825
CDD2 $\left(\sigma =5\right)$	64.1485	56.7222
CDD2 $\left(\sigma =20\right)$	89.0010	82.0980
